# Deeply Pigmented Reticulated Acanthoma with Sebaceous Differentiation Mimicking Cutaneous Malignancy: A Case Report and Review of the Literature

**DOI:** 10.3390/dermatopathology13010004

**Published:** 2025-12-30

**Authors:** Padol Chamninawakul, Xiaotian Wu, Joyce S. S. Lee

**Affiliations:** 1 National Skin Centre, Singapore 308205, Singapore; wu.xiaotian@nhghealth.com.sg (X.W.); joycelee@nhghealth.com.sg (J.S.S.L.); 2 Institute of Pathology, Department of Medical Services, Ministry of Public Health, Bangkok 10400, Thailand

**Keywords:** reticulated acanthoma with sebaceous differentiation, RASD, sebaceous neoplasm, melanoma mimicker, diagnostic pitfall

## Abstract

Reticulated acanthoma with sebaceous differentiation (RASD) is a rare, benign cutaneous neoplasm. Its variable clinical presentation frequently mimics both benign and malignant entities, posing a significant diagnostic challenge. We report a case of pigmented RASD in a 78-year-old Malay male of Fitzpatrick skin type IV who presented with a 5-year history of an 8 × 5 mm deeply pigmented, asymmetrical nodule on the left upper back, with a 2 mm central raised area showing less pigmentation. The lesion was clinically suspicious for malignant melanoma. Histopathological examination revealed characteristic features of RASD: a broad, plate-like, reticulated and pigmented epidermal proliferation with clusters of mature sebocytes at the bases of anastomosing rete ridges. Following biopsy confirmation, the residual lesion is being managed conservatively with observation. This case demonstrates an unusual heavily pigmented clinical presentation that completely obscured the typical yellowish hue associated with sebaceous differentiation, highlighting pigmented RASD as an important diagnostic pitfall in patients with skin of color. In conclusion, RASD should be included in the differential diagnosis of pigmented cutaneous lesions, especially in patients with skin of color. Recognition of this benign entity can prevent unnecessary aggressive surgical intervention.

## Simple Summary

Reticulated acanthoma with sebaceous differentiation is a rare, benign skin tumor characterized by the presence of sebaceous gland cells within a net-like growth pattern. A yellowish hue, when present, may provide a clinical clue to the underlying sebaceous gland differentiation. However, in patients with darker skin tones, heavy pigmentation can completely obscure this characteristic appearance. We report a case in an elderly patient whose deeply pigmented skin lesion raised clinical concern for malignant melanoma. Microscopic examination revealed the true benign diagnosis. This case highlights an important diagnostic pitfall: in patients with skin of color, this benign tumor can masquerade as skin cancer due to excessive pigmentation. Recognizing this unusual presentation is crucial for dermatologists and pathologists to avoid unnecessary aggressive surgical treatment and to provide appropriate reassurance to patients.

## 1. Introduction

Reticulated acanthoma with sebaceous differentiation (RASD) is a rare, benign cutaneous neoplasm first described by Steffen and Ackerman in 1994 [1] and subsequently formally characterized by Fukai et al. in 2006 [2]. This entity is characterized histopathologically by a distinctive combination of reticulated epidermal proliferation resembling seborrheic keratosis and clusters of mature sebocytes at the bases of anastomosing rete ridges [2,3,4].

Due to its rarity and variable clinical presentation, RASD is likely underdiagnosed, often being misclassified as seborrheic keratosis, eccrine poroma, or other sebaceous neoplasms [2,3,4]. Clinically, RASD can mimic a spectrum of cutaneous lesions ranging from benign entities, such as seborrheic keratosis to malignancies including melanoma, Bowen disease, and basal cell carcinoma (BCC), making histopathological examination essential for definitive diagnosis [5,6].

The entity has been described under various synonyms, including superficial epithelioma with sebaceous differentiation, sebocrine adenoma, poroma with sebaceous differentiation, and seborrheic keratosis with sebaceous differentiation [4,5]. This nomenclatural inconsistency has contributed to diagnostic confusion and likely underreporting.

The potential association between RASD and Muir–Torre syndrome (MTS), a variant of Lynch syndrome characterized by sebaceous neoplasms and visceral malignancies, remains controversial. Although most reported cases show intact mismatch repair (MMR) protein expression, at least one case demonstrated MMR protein loss, causing some authors to advocate evaluation of all cases of RASD for microsatellite instability [7,8].

We present a case of pigmented RASD in an elderly Malay male presenting with an unusually deeply pigmented lesion clinically suspicious for malignant melanoma. This case highlights the entity’s potential to present as a deeply pigmented neoplasm in patients with skin of color with heavy melanization obscuring its typical yellowish clinical appearance.

## 2. Case Description

A 78-year-old Malay male of Fitzpatrick skin type IV presented with a pigmented lesion on the left upper back that he had noticed for approximately 5 years. The patient had not paid significant attention to the lesion due to its location. He reported occasional mild pruritus but denied pain, bleeding, or rapid growth. His medical history was unremarkable, with no personal or family history of internal malignancies or cutaneous sebaceous neoplasms.

**Figure 1:**
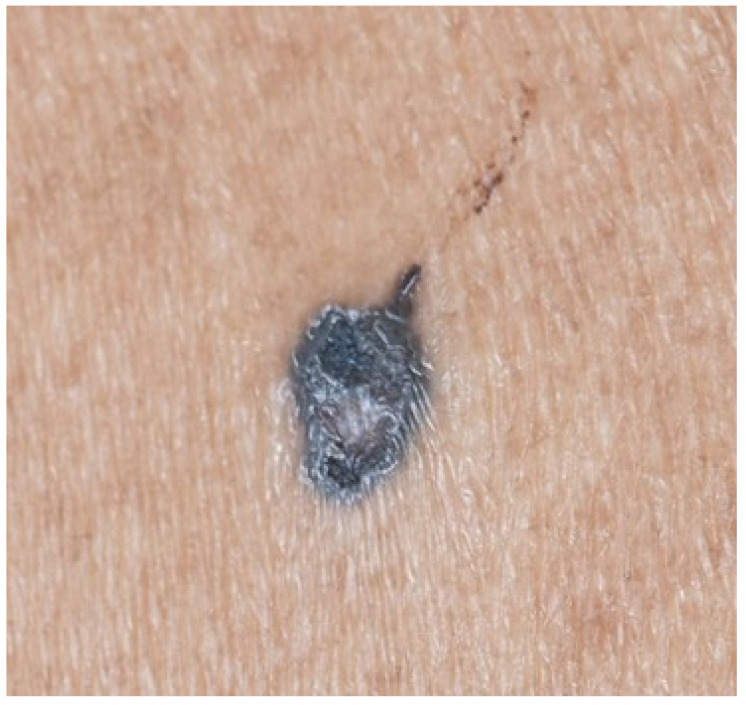
Clinical photograph of reticulated acanthoma with sebaceous differentiation. A well-demarcated, asymmetrical, deeply pigmented nodule (8 × 5 mm) on the left upper back. The lesion displays dark gray to blue-black coloration with a central raised area (2 mm) showing less pigmentation, surrounded by a deeply pigmented rim. This was concerning clinically for malignant melanoma. Note the complete absence of the yellowish hue typically associated with sebaceous neoplasms.

Clinical examination revealed a solitary, well-demarcated, asymmetrical nodule measuring 8 × 5 mm on the left upper back (Figure 1). The lesion was deeply pigmented, displaying a striking dark gray to blue-black coloration with irregular but sharply defined borders. A central raised papular component measuring approximately 2 mm was noted, with light gray pigmentation that contrasted markedly with the surrounding darker pigmentation. On dermoscopy, the deeply pigmented area exhibited black pigment globules, but the lighter central papule lacked similar features. There were no features of a basal cell carcinoma such as arborizing blood vessels. Notably, no yellowish structures suggestive of sebaceous differentiation were identified on dermoscopy. The dermoscopic findings, dominated by black pigment globules without specific features of either sebaceous neoplasm or BCC, were suggestive of a melanocytic lesion. The asymmetric distribution of these globules between the pigmented periphery and lighter center further heightened suspicion for malignant melanoma.

A punch biopsy was performed for histopathological evaluation.

**Figure 2:**
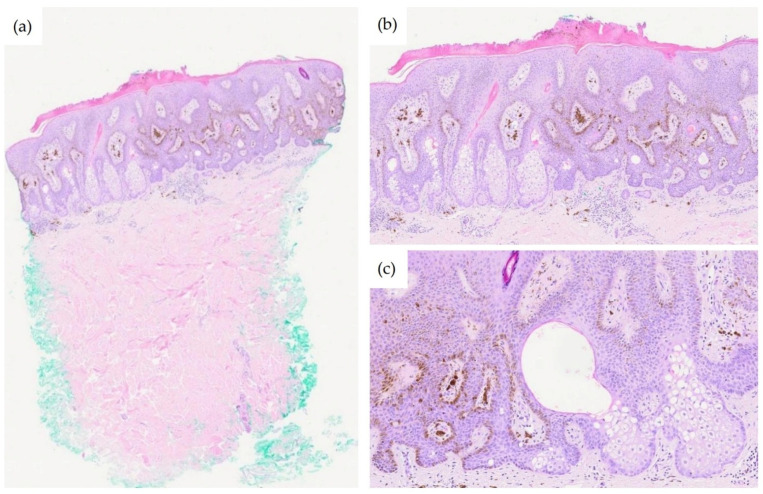
Histopathological features of reticulated acanthoma with sebaceous differentiation. (**a**) Scanning magnification showing a superficial epithelial proliferation with a broad, plate-like, reticulated architecture and anastomosing rete ridges; (**b**) Low-power view demonstrating clusters of mature sebocytes at the bases of the anastomosing epithelial cords. Note the prominent melanin pigment within the keratinocytes and melanophages within the papillary dermis; (**c**) Medium-power view showing mature sebocytes with characteristic multivacuolated cytoplasm and centrally located, scalloped nuclei, surrounded by a thin rim of germinative basaloid cells. Sebaceous ductal differentiation is present.

Microscopic examination revealed a superficial epithelial proliferation exhibiting a broad, plate-like architecture with a reticulated (net-like) pattern of anastomosing rete ridges extending into the superficial reticular dermis (Figure 2a). The proliferating epithelium was composed of bland-appearing squamous cells with basaloid cells at the periphery. No cytological atypia, increased mitotic activity, or necrosis were identified.

The defining histopathological feature was the presence of clusters of mature sebocytes, characterized by their multivacuolated cytoplasm and centrally located, scalloped nuclei, situated at the bases of the anastomosing epithelial cords, in association with sebaceous ductal differentiation (Figure 2b,c). The sebocytes were surrounded by a thin rim of germinative basaloid cells. Melanin pigment was prominently distributed throughout the lesion, both within keratinocytes and as melanin incontinence in the papillary dermis, correlating with the deeply pigmented clinical appearance. No connection to pilosebaceous units was identified.

Based on the characteristic histopathological constellation of reticulated epidermal proliferation with sebocyte aggregates at the bases of anastomosing rete ridges, a diagnosis of pigmented reticulated acanthoma with sebaceous differentiation (RASD) was rendered.

Following histopathological confirmation of RASD, a benign neoplasm, the patient was seen for follow-up. At the 2-week follow-up visit, clinical examination revealed a residual rim of dark brown lesion at the biopsy site. Given the confirmed benign nature of RASD, a conservative approach with clinical observation was elected.

## 3. Discussion

Reticulated acanthoma with sebaceous differentiation represents one of the rarest entities in the spectrum of cutaneous neoplasms with sebaceous differentiation [7,8,9]. Our case contributes to this limited body of knowledge by demonstrating an atypical, heavily pigmented clinical presentation that drew concerns clinically for a cutaneous malignancy such as malignant melanoma (Table 1).

### 3.1. Clinical and Dermoscopic Considerations

The clinical presentation of RASD is variable and overlaps with both benign and malignant cutaneous neoplasms. Most reported cases describe yellowish, brownish, or reddish papules or plaques, with the yellowish hue often providing a clinical clue to underlying sebaceous differentiation [3,9]. In contrast, our case presented with a deeply pigmented, dark gray to blue-black lesion, completely lacking the characteristic yellowish tint (Figure 1). In patients with skin of color, cutaneous neoplasms can be heavily pigmented with melanin. This proves a potential pitfall in diagnosis for the clinician, both from the rarity of RASD itself and its even rarer pigmented presentation.

The prominent central area of hypopigmentation within our case further heightened concern for malignancy, as this appearance can simulate regression in melanoma. The 5-year indolent course was reassuring, but insufficient to exclude malignancy without histopathological confirmation.

Dermoscopy may reveal yellowish structures corresponding to sebocyte aggregates [3,6,10]. Ito et al. described linearly or reticularly arranged bright yellow dots as a characteristic dermoscopic clue [10]. Ogawa et al. reported dermoscopic findings of a white-yellowish reticular structure with scattered dotted vessels in their case [11]. However, when heavy pigmentation obscures the yellowish component, the dermoscopic appearance can closely simulate melanoma with regression, featuring gray structures and white reticular areas [5].

### 3.2. Histopathological Differential Diagnosis

The histopathological diagnosis of RASD relies on recognizing its distinctive combination of features: a broad, superficial, reticulated epidermal proliferation with organized clusters of mature sebocytes at the bases of anastomosing rete ridges. The key histological differential diagnoses include:

Seborrheic keratosis with sebaceous differentiation: While both entities show acanthosis and may contain sebocytes, seborrheic keratosis lacks the extensive, organized sebaceous component characteristic of RASD. In RASD, sebocyte clusters are consistently found at the bases of anastomosing rete ridges in a band-like pattern, whereas in seborrheic keratosis, sebaceous differentiation is focal and haphazard [4,9].

Sebaceous adenoma: This entity shows sharply demarcated, perpendicularly oriented lobules of sebocytes surrounded by basaloid germinative cells. The reticulated epidermal proliferation characteristic of RASD is absent [7].

Eccrine and apocrine poroma: While both poromas and RASD may show anastomosing cords of epithelial cells, and apocrine poromas may show sebaceous differentiation, poromas are composed of poroid and cuticular cells with ductal differentiation [3].

Nevus sebaceus: This hamartomatous lesion typically presents at birth or early childhood with papillomatous epidermal hyperplasia, loss of mature hair follicles which are replaced by primitive hair germs, and ectopic apocrine glands. The large, hyperplastic sebaceous glands of a nevus sebaceus lack the organized reticulated pattern of an RASD [3,4,7].

### 3.3. Muir–Torre Syndrome Considerations

The relationship between RASD and Muir–Torre syndrome (MTS) remains controversial but clinically important. Most reported RASD cases have shown intact expression of MMR proteins (MLH1, MSH2, MSH6, PMS2) on immunohistochemistry, suggesting a sporadic nature without MTS association [3,5,12]. However, Shon et al. reported a case demonstrating reduced MSH6 expression, supporting a potential association with MTS in some cases [8]. In our case, MMR immunohistochemistry was not performed. The patient had no personal or family history of internal malignancies or multiple sebaceous neoplasms, and no clinical features suggestive of MTS.

### 3.4. Management Considerations

Complete surgical excision has been the standard management approach in most reported cases of RASD [5,7]. As the lesion is benign, conservative management with observation of residual lesion is reasonable following histopathological confirmation.

### 3.5. Potential for Malignant Transformation

Although RASD is considered a benign neoplasm, Yoshida et al. reported a case of Bowen disease (squamous cell carcinoma in situ) arising within a RASD lesion 11 years after initial presentation in a 65-year-old man [13]. This case raises the possibility, albeit rare, of malignant transformation within RASD and supports complete excision when feasible. Long-term follow-up is recommended for patients managed conservatively.

## 4. Conclusions

This case highlights pigmented RASD as a diagnostic pitfall that can mimic melanoma in patients with skin of color. Histopathological examination remains essential for accurate diagnosis and appropriate management.

## Figures and Tables

**Table 1:** Comparison of the present case with published RASD cases [2,3,4,5,6,7,8,9,10,11,12,13].

Feature	Published Cases	Present Case	Concordance
Age	52–93 years (Mean 68 years)	78 years	Concordant
Sex	Male 8, Female 4	Male	Concordant
Number of lesions	Solitary in all cases	Solitary	Concordant
Location	Back, trunk, lower body (buttocks, hip), forearm, chest	Upper back	Concordant
Size	6 mm–50 mm	8 × 5 mm	Concordant
Duration	2 months to 30 years	~5 years	Concordant
Symptoms	Asymptomatic or pruritic	Mild pruritus	Concordant
Clinical color	Yellow, brown, red, white, variegated	Dark gray to black	Atypical
Clinical differential	Seborrheic keratosis, Bowen disease, Basal cell carcinoma, Melanoma	Melanoma	Concordant

## Data Availability

The original contributions presented in this study are included in the article. Further inquiries can be directed to the corresponding author.
